# Harnessing selenocysteine reactivity for oxidative protein folding†
†Electronic supplementary information (ESI) available. See DOI: 10.1039/c4sc02379j


**DOI:** 10.1039/c4sc02379j

**Published:** 2014-09-23

**Authors:** Norman Metanis, Donald Hilvert

**Affiliations:** a Laboratory of Organic Chemistry , ETH Zürich , 8093 Zürich , Switzerland . Email: hilvert@org.chem.ethz.ch ; Fax: +41-44-632-1486

## Abstract

Although oxidative folding of disulfide-rich proteins is often sluggish, this process can be significantly enhanced by targeted replacement of cysteines with selenocysteines. In this study, we examined the effects of a selenosulfide and native *versus* nonnative diselenides on the folding rates and mechanism of bovine pancreatic trypsin inhibitor. Our results show that such sulfur-to-selenium substitutions alter the distribution of key folding intermediates and enhance their rates of interconversion in a context-dependent manner.

## Abstract 2

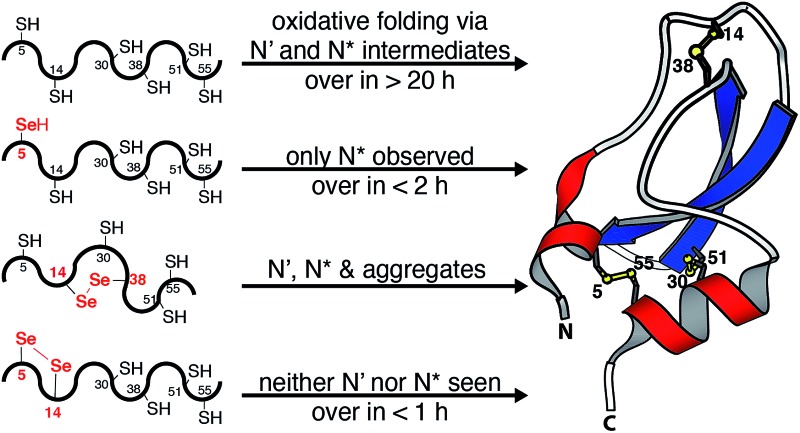
Turbo-charged folding with selenium: targeted replacement of cysteines in proteins with selenocysteines is a valuable strategy for increasing the rates of oxidative protein folding, altering folding mechanisms, and rescuing kinetically trapped intermediates.

## 

Proper folding is critical to protein function. Most proteins fold spontaneously to their native three-dimensional structure guided by information encoded in the primary amino acid sequence.^1^ For proteins that contain multiple disulfide bonds, this process is complicated by the need for additional oxidation, reduction, and rearrangement steps.^2^ Because the number of possible disulfide crosslinks increases factorially with the number of cysteines, formation of scrambled disulfide bond isomers and accumulation of kinetically trapped intermediates may limit both folding rates and yields.

Redox buffers—typically mixtures of oxidized and reduced glutathione (GSSG and GSH, respectively)—have been used extensively to enhance oxidative protein folding *in vitro*.^3–5^ Recently, small molecule diselenides such as selenoglutathione (GSeSeG) were shown to possess significant advantages over disulfides in such reactions, affording faster rates and higher yields for many disulfide-rich proteins.^6,7^ Both thiol oxidation and subsequent disulfide bond shuffling are facilitated by the higher polarizability of selenium compared to sulfur and the greater acidity of selenols *versus* thiols (Δp*K*_a_ ≈ 3).^8,9^ Because selenols are readily oxidized, such processes can even be performed with catalytic amounts of diselenide.^10^

Intramolecular catalysis of oxidative protein folding is also possible if cysteine residues in a protein are replaced by selenocysteine (abbreviated as Sec or U).^11–17^ Typically, selenocysteines are placed at positions that would yield native crosslinks. Because diselenides are more stable than selenosulfides and disulfides, diselenide-bond formation provides an effective means of generating specific crosslinks. In addition to increasing folding rates by up to two orders of magnitude, this strategy affords isosteric variants of the native protein that can be more resistant to reduction and disulfide scrambling.^18^ Nonnative diselenide crosslinks have been similarly exploited to trap kinetically unstable protein folding intermediates^19^ and to bias early folding events.^20^ For example, we showed that nonnative connectivities could substantially improve the folding efficiency of bovine pancreatic trypsin inhibitor (BPTI, Fig. 1) by altering the balance of productive *versus* non-productive folding routes.^20^

**Fig. 1:**
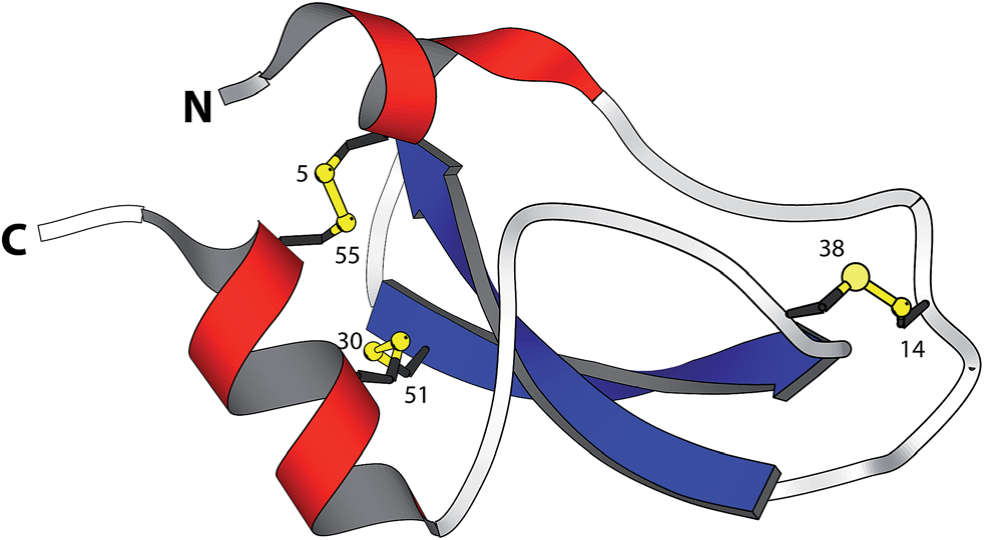
Structure of BPTI. The folded protein contains three native disulfide bonds between cysteines 5–55, 14–38, and 30–51 (PDB entry: 1BPI).

BPTI is a 58-residue protein stabilized by three disulfide bonds. It normally folds by a bifurcated pathway characterized by a small number of intermediates containing one and two native disulfide bonds (Fig. 2a).^21–23^ These include two relatively long-lived species, N* ([5–55; 14–38]) and N′ ([14–38; 30–51]), which must undergo partial unfolding and rate-limiting disulfide bond rearrangements to reach the native state (N). To perturb the normal steady-state distribution of intermediates, we replaced both Cys5 and Cys14 with selenocysteine.^20^ Formation of the nonnative 5–14 crosslink accelerated folding by altering the population of one-disulfide intermediates and eliminating the kinetically trapped two-disulfide intermediates (Fig. 2b). In the current study, we have prepared and characterized two additional selenoproteins to gain further insight into the influence of selenium on the BPTI folding mechanism. The first contains a single selenocysteine at position 5, whereas the second introduces a diselenide in place of the native 14–38 disulfide bond.

**Fig. 2:**
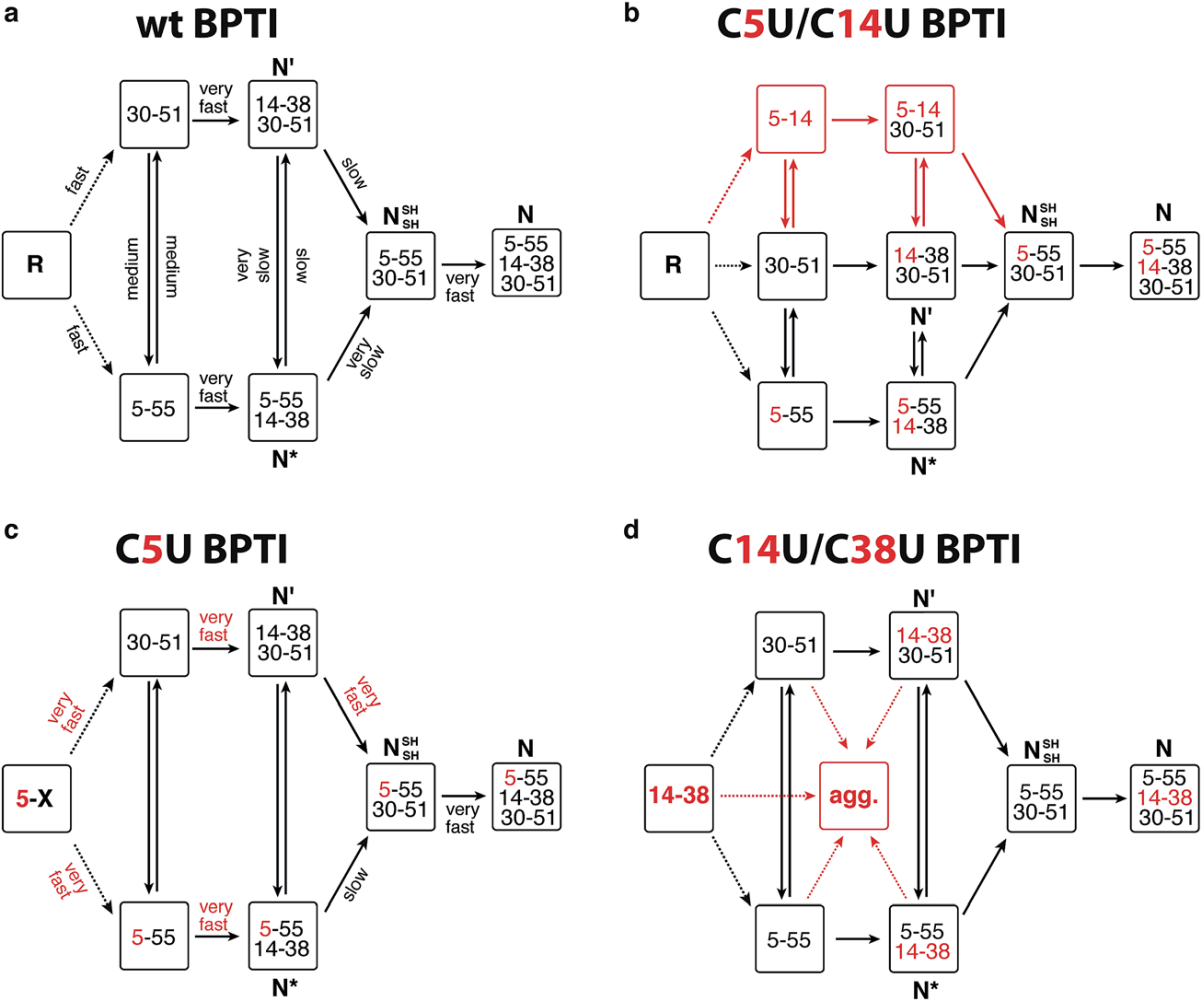
Folding mechanisms of BPTI analogs. (a) Kinetically favored folding pathway for wt BPTI.^23^ R refers to the fully reduced protein; intermediates that accumulate during folding are indicated by the disulfide bonds they contain. Initial oxidation of R affords a broad distribution of single disulfide intermediates (dotted lines) that rearrange to [5–55] and [30–51].^24–26^ Qualitative estimates of the relative rates of individual steps are indicated. (b) C5U/C14U BPTI can fold *via* the normal BPTI mechanism (black) or by a new reaction channel (red) that avoids the long-lived N* and N′ intermediates;^20^ (c) The observation of N* suggests that C5U BPTI folds by the standard BPTI pathway, but the presence of selenocysteine increases the reactivity of both N* and N′; (d) The folding pathway for C14U/C38U BPTI is similar to that of wt BPTI, but the reactivity of the 14–38 diselenide promotes extensive precipitation.

The C5U BPTI variant was chemically synthesized by a previously described three-fragment native chemical ligation strategy.^20,27,28^ To remove impurities that might affect folding kinetics, the crude ligation product was first folded under aerobic conditions and purified as a single species by HPLC. Following reduction with DTT and rapid reoxidation of the selenol by air, the selenoprotein was isolated by HPLC as a mixture of isomers that possess a single 5–X selenosulfide and four reduced thiols. This species folded rapidly under anaerobic conditions at pH 8.7 using glutathione as an oxidant (Fig. 3a). In contrast to wt BPTI, which requires >21 h to convert completely to N (Fig. 3b), the entire process was finished within 3 h. In fact, more than half of the protein reached the native state within one minute. Approximately 35% of the sample formed a transient intermediate with a retention time of 24.6 min, similar to that of N* in the natural folding pathway. Mass spectrometric analysis of this species confirmed that it contained only two crosslinks (either two disulfides or one disulfide plus a selenosulfide), whereas tryptic mapping provided evidence for an N*-like structure (see ESI†). Although cleavage of the 5–55 crosslink is not required for conversion of this intermediate to N, C5U N* reacted faster than its wild-type counterpart, perhaps because the lability of the selenosulfide facilitated its equilibration with N′ (Fig. 2c). The presence of a free selenol in C5U N′ would be expected to promote intramolecular rearrangement to C5U NSHSH, explaining why this species did not accumulate during folding. As previously seen for wt BPTI (Fig. 3d), utilization of GSeSeG instead of GSSG as the oxidant further accelerated the N* → N conversion (Fig. 3c).^29^ The rate enhancement (∼2-fold) was relatively modest at pH 8.7 (Fig. 3f), but experiments with C5U/C14U BPTI suggest that even larger effects should be attainable at lower pH where oxidative folding is inherently slower.^20^

**Fig. 3:**
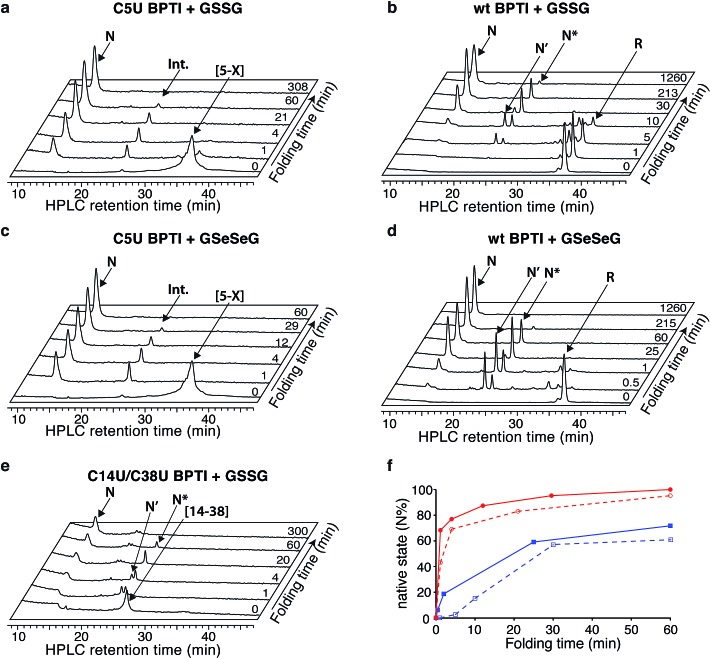
Anaerobic folding of BPTI variants at pH 8.7. Representative HPLC chromatograms of acid-quenched aliquots from folding reactions with (a) C5U BPTI with GSSG, (b) wt BPTI with GSSG; (c) C5U BPTI with GSeSeG; (d) wt BPTI with GSeSeG; and (e) C14U/C38U BPTI with GSSG. (f) Kinetic traces of the initial stages of the folding reactions of wt BPTI (blue) and C5U BPTI (red), in the presence of GSSG (dashed) or GSeSeG (solid). [protein] = 30 μM; [GSSG], [GSeSeG] = 150 μM.

The C14U/C38U analog is interesting because of the central role played by the native 14–38 disulfide in BPTI folding (Fig. 1).^21–23^ Kinetic studies on the wt protein have shown that 14–38 is the first disulfide bond reduced during reductive unfolding;^30^ it is also the first formed during oxidative folding, but rapidly rearranges to the one-disulfide intermediates [5–55] and [30–51].^24–26^ Reinstallation of the 14–38 crosslink then affords N* and N′, and subsequent cleavage of this bond in the thiol-disulfide interchange reactions leading to the native state is rate limiting overall.^21–23^ To examine how replacement of the 14–38 disulfide with a diselenide affects this complex reaction manifold, we synthesized C14U/C38U BPTI by a route analogous to that used to prepare the C5U and C5U/C14U analogs. The selenoprotein was isolated as a single peak by HPLC, albeit in lower yield than C5U/C14U BPTI. High-resolution mass spectrometric analysis indicated the presence of a single crosslink, consistent with selective formation of the 14–38 diselenide.

The low yield obtained for C14U/C38U BPTI can be attributed to nonspecific aggregation and precipitation of the protein, which also complicated the folding experiments. In contrast to the other BPTI analogs, this variant gave considerable amounts of white precipitate under standard anaerobic folding conditions (30 μM protein, 150 μM GSSG, pH 8.7). Use of GSeSeG as oxidant did not ameliorate this problem. Although HPLC chromatograms of reaction aliquots had relatively low peak intensities, reflecting the losses to precipitation, folding of the residual soluble protein could be monitored nonetheless. The data (Fig. 3e) indicate that C14U/C38U BPTI reached the native state almost as fast as C5U/C14U and C5U BPTI. In this case, though, two transient intermediates with retention times of 24 and 24.7 min were observed, which likely correspond to N′ and N* based on their retention times and relative reactivities (N′ > N*). Although replacing the native 14–38 disulfide in these species with a thermodynamically more stable diselenide could conceivably have inhibited folding, both intermediates were efficiently converted to N. Reactions of thiols with diselenides can be orders of magnitude faster than analogous thiol-disulfide exchange reactions,^9^ and the exposed nature of the 14–38 diselenide bond likely further facilitated the glutathione-dependent interchange reactions required to access the native state. Nevertheless, the low overall yields of final protein and the accumulation of various misfolded species (19–21 min) (Fig. 3e) offset the kinetic advantage over wt BPTI.

Targeted replacement of cysteines by selenocysteines in proteins represents a simple strategy for modulating the kinetics and thermodynamics of oxidative folding pathways.^12,31,32^ The utility of diselenides for steering this process and increasing both rates and yields is now well established for several systems. In most studies diselenides have been used as surrogates for native disulfide bonds, but such sites need not provide the greatest benefit. In BPTI, the nonnative 5–14 diselenide crosslink produced higher yields of folded protein^20^ than the native 14–38 diselenide, despite the presence of two relatively unstable Se–S bonds in the final product. Aggregation and precipitation of the C14U/C38U analog are likely due to the solvent accessibility and inherent reactivity of the 14–38 crosslink. This hypothesis finds support in studies on conotoxins, showing that yields of native protein were lower when solvent-exposed rather than buried disulfides were replaced with diselenides.^13,16,17^

The results obtained with C5U BPTI show that beneficial effects on folding are not restricted to diselenides. Unlike the nonnative 5–14 diselenide, the single selenocysteine does not appear to alter the normal folding pathway. Nevertheless, it accelerates folding to a similar extent, in this case by increasing the reactivity of the kinetically trapped N* and N′ species that normally limit folding efficiency. Additional work will be needed to establish the generality of this finding. However, since single selenocysteines can be introduced into proteins ribosomally,^33–35^ such reactivity could be advantageous for the biotechnological production and folding of diverse cysteine-rich therapeutic proteins.
